# Etiological Profile of First Episode Seizures in Paediatric Patients at a Tertiary Care Centre: A Descriptive Cross-sectional Study

**DOI:** 10.31729/jnma.8535

**Published:** 2024-04-30

**Authors:** Madhu Shah, Saurav Poudel, Bivusha Parajuli, Niranjan KC, Rumi KC

**Affiliations:** 1 Department of Paediatrics, Nobel Medical College Teaching Hospital, Biratnagar, Morang, Nepal; 2 Department of Internal Medicine, Nobel Medical College Teaching Hospital, Biratnagar, Morang, Nepal; 3 Nepal Medical College and Teaching Hospital, Attarkhel, Kathmandu, Nepal

**Keywords:** *febrile seizures*, *paediatrics*, *seizures*

## Abstract

**Introduction:**

Seizures are the most common neurological emergency and one of the most common reasons for paediatrics hospital admissions. This study aimed to identify the etiology, clinical profile, and immediate outcome of children with the first episode of seizure in Eastern Nepal.

**Methods:**

This was a prospective descriptive study carried out in the Tertiary Care Centre from September 2022 to August 2023. Ethical clearance was taken from the Institutional Review Committee (Ref no:654/2022). Convenience sampling was done to include 170 children presenting with the first episode of seizure at age 6 months to 15 years. Variables collected were demographics, clinical presentations, family history, trauma history, laboratory tests, neuroimaging, EEG, final diagnosis, and immediate outcome.

**Results:**

A total of 170 patients were admitted with the first episode of seizure with 123 (72.36%) males and 47 (27.64%) females. The mean age of the patients was 5.13±2.95 years with 104 (61.18%) patients under 5 years of age. The most common seizure was generalized tonic-clonic type in 132 (77.64%) patients. The most common associated symptom was fever in 150 (88.23%) children. Neuroimaging was abnormal in 52 (30.59%) patients, with neurocysticercosis seen in 27 (15.88%). The most common etiology was febrile seizure in 92 (54.17%) patients, neurocysticercosis in 27 (15.88%), and meningitis in 12 (7.05%).

**Conclusions:**

Febrile seizures, neurocysticercosis, infection, and trauma were the major causes of seizures in children. When simple febrile seizures were unlikely, lumbar puncture, neuroimaging, and laboratory tests were useful tools for diagnosing etiologies of seizures.

## INTRODUCTION

Seizures are disturbances of neurological function due to excessive, hypersynchronous discharge of neurons in the brain.^1^ Seizures are the most common neurological paediatrics emergency.^2,3^ Seizure incidence is reported to be between 4-10% of all children under 16 years and around 1% of all emergency visits.^2,4,5^ The common causes of the first episode of seizure in children include fever, infection, developmental or neurological disorders, traumatic head injury, and metabolic abnormalities. Several studies worldwide conclude that febrile seizures are the most common type of seizure seen in children.^3,4,6^ CNS infections are the most common acquired cause of seizure in the developing world.^3,5,7^

The proper management of seizures in children is always challenging, more so in resource-limited countries like Nepal due to the lack of proper investigations, trained personnel, and economic hardships. There is a lack of adequate evidence-based studies about the etiology and outcome of seizures in children from Nepal. Through this study, we seek to contribute to the existing literature and evidence-based medicine about the etiological profile of the first episode of seizure in children.

This study aimed to identify the clinical presentation, etiology, necessity of added investigations, and immediate outcome of the first episode of seizure in children admitted to tertiary care centers, and add to the prevalent literature to increase evidence-based medicine practices.

## METHODS

This was a descriptive, cross-sectional study done by collecting data from 1 September 2022 to 31 August 2023. In this research, children who were admitted to a tertiary hospital after experiencing their first episode of seizure were studied. Ethical approval was taken from the Institutional Review Committee of Nobel Medical College Teaching Hospital (Reference number: 654/2022). Data was collected after obtaining written informed consent from the legal guardians of all the children included in the study, following a thorough explanation of the nature and purpose of the study. All children admitted with new onset seizures between 6 months to 15 years of age at Nobel Medical College Teaching Hospital, Biratnagar, Morang, Nepal were included in the study. Children previously diagnosed with epilepsy/seizure disorder and/or taking anti-epileptic drugs were excluded from the study.

Variables like age, sex, type of seizure (generalized tonic-clonic, partial, absence, myoclonic, status, others), associated symptoms (fever, cough, rhinorrhea, vomiting, diarrhea, headache), family history of seizure or epilepsy, neurodevelopmental history, history of head trauma, laboratory test, EEG, and neuroimaging were studied during the study. A convenience sampling technique was used for data collection. The sample size was calculated using the following formula:


$$\begin{array}{ll} \text{n} & ={\text{Z}}^{2}\times \frac{\text{p}\times \text{q}}{{\text{e}}^{\text{2}}}\\ & =1.96^{2}\times \frac{0.127\times 0.873}{0.06^{2}}\\ & =119 \end{array}$$

Where,

n = minimum required sample size
Z = 1.96 at 95% Confidence Interval (CI)
p = proportion of children getting admitted with the first episode of seizure taken as 12.7% ^5^
q = 1-p
e = margin of error, 6%

The calculated sample size was 119; however 170 patients were included in the study.

Simple febrile seizure was defined as a single occurrence of seizure in the absence of central nervous system infection and other possible causes of seizure. Recurrent febrile seizures were recurrence of febrile seizures in the same patient within 24 hours. All seizures were evaluated and classified according to the International League Against Epilepsy (ILAE) guidelines.^8^

Neuroimaging was done for all patients regardless of the etiology of the seizure to rule out the possibility of obscure central nervous system causes of seizure such as congenital lesions, mass lesions, and neurocysticercosis among others. Neuroimaging preceded lumbar puncture in most cases. Lumbar puncture was indicated for cases where the cause of seizures was of doubt, after ruling out structural abnormalities through neuroimaging. Lumbar puncture was done in an aseptic manner after ruling out increased intracranial pressure (ICP) through neuroimaging and/or fundus examination in older age group paediatrics patients. Lumbar puncture was also done for all patients under one year of age according to the American Academy of Paediatrics (AAP) Guidelines for the workup of febrile seizures.^9^

The data were entered in a Microsoft Excel and statistical analysis was done with IBM SPSS Statistics.

## RESULTS

Over the study period of 1 year, 1819 patients were admitted to the paediatrics ward and paediatrics ICU of Nobel Medical College Teaching Hospital, Biratnagar, Nepal. Among them, 219 patients (12.03%) presented with the first episode of seizure. Of the 219 patients, 170 patients were included in the study by cumulative sampling method.

Generalized tonic-clonic seizure was the most common type of seizure observed during our study. It was observed in 132 (77.64%) patients. Other types of seizures observed were partial seizure in 4 (2.36%) patients, absence seizure in 2 (1.18%) patients, myoclonic seizure in 4 (2.36%) patients, status epilepticus in 14 (8.23%), and other types of seizures were seen in 14 (8.23%) patients (Table 1).

**Table 1:** Presentation of seizures (n = 170).

Type of seizure	n (%)
GTCS	132 (77.64)
Partial	4 (2.36)
Absence	2 (1.18)
Myoclonic	4 (2.36)
Status epilepticus	14 (8.23)
Other	14 (8.23)

The mean age of the patients admitted with seizure was 5.13±2.95 years. Out of 170 patients 104 (61.18%) of patients were under 5 years of age, 41 (24.12%) were between 5 years and 10 years of age and 25 (14.70%) were between 10 years and 15 years of age. Out of 170 patients, 123 (72.36%) patients were male, while 47 (27.64%) were female.

Along with seizures, the patients also presented with other symptoms. Fever was present in 150 (88.23%) patients, cough was present in 44 (25.88%) patients, rhinorrhea was present in 12 (7.05%) patients, vomiting was present in 68 (40%) patients, diarrhea was present in 5 (2.94%) patients, and headache was present in 40 (23.52%) patients. A positive family history of seizure was present in 13 (7.64%) patients. Eleven (6.47%) patients were admitted with posttraumatic seizures. One hundred thirty-seven (93.8%) patients had normal developmental history while 9 (5.29%) had abnormal developmental history (Table 2).

**Table 2:** Associated symptoms and relevant history (n = 170).

Variables	n (%)
**Associated symptoms**	
Fever	150 (88.23)
Cough	44 (25.88)
Rhinorrhea	12 (7.05)
Vomiting	68 (40)
Diarrhea	5 (2.94)
Headache	40 (23.52)
**Associated history**	
Family history of seizure	13 (7.64)
History of trauma	11 (6.47)
Abnormal developmental history	9 (5.29)

Laboratory analysis of blood was done for all cases. Cerebro-spinal fluid (CSF) was done for all cases under the age of one year according to the AAP guidelines and of those patients whose seizures presented as unlikely of febrile seizures or could not easily be classified as febrile seizures, after ruling out the risk of increased ICP by neuroimaging or fundus examination. On blood examination, more than half of the patients 93 (54.71%) had a raised leukocyte count while the remaining 77 (45.29%) patients had normal leukocyte count. Raised C-reactive protein (CRP) levels were seen in 121 (71.18%) patients. Abnormal electrolyte levels were seen in 28 (16.47%) patients. Very few patients 9 (5.29%) presented with hypoglycemia. Positive blood culture was seen in 4 (2.35%) patients. CSF analysis of patients was abnormal in 46 (27.05%) patients (Table 3).

**Table 3:** Laboratory findings (n= 170).

Variables	n (%)
**Blood examination**	
**Leukocyte count**	
Raised	93 (54.71)
Normal	77 (45.29)
**CRP levels**	
Raised	121 (71.18)
Normal	49 (28.82)
**Abnormal electrolyte levels**	28 (16.47)
Hypoglycemia	9 (5.29)
Hyponatremia	7 (4.11)
Hypokalemia	4 (2.35)
Hypocalcemia	4 (2.35)
Hypernatremia	3 (1.76)
Hyperkalemia	1 (0.58)
**Positive blood culture**	**4 (2.35)**
**CSF examination** ^*^	
Abnormal CSF results	46 (27.05)
Bacterial meningitis	12 (7.05)
Isolated increased protein	10 (5.88)
Xanthochromia	8 (4.71)
Isolated decreased glucose	7 (4.11)
Viral meningoencephalitis	5 (2.94)
Increased eosinophils	4 (2.35)

* Lumbar puncture was done in age group less than 1 year, finding of the lumbar puncture is calculated in total population.

Neuroimaging of all cases was performed for all the cases regardless of etiology to rule out the possibility of missing obscure causes of seizures such as congenital defects, mass lesions, neurocysticercosis, and others, due to the high prevalence of neurocysticercosis in our region. Among all the cases, 52 (30.59%) patients presented with abnormal radiological scans. Among the abnormal neurological scans, neurocysticercosis was seen in 27 (15.88%) patients. Intracranial hemorrhage was seen in 9 (5.29%) patients and brain tumor was seen in 2 (1.18%) patients. In neuroimaging, signs of a developmental defect were observed in 14 (8.23%) patients. EEG was done for the cases where the diagnosis of febrile seizures and definite diagnosis from neuroimaging was not clear, and where epileptic syndromes were suspected. Of all the patients, EEG was performed for 31 cases (18.23%), which showed positive EEG findings of seizure disorder in 9 (5.29%) patients (Table 4).

**Table 4:** Neuroimaging and EEG (n = 170).

Variables	n (%)
Neuroimaging	
Normal	118 (69.41)
Neurocysticercosis	27 (15.88)
Intracranial hemorrhage	9 (5.29)
Brain tumor	2 (1.18)
Developmental brain defect	14 (8.23)
**EEG**	
Not performed	139 (81.77)
Performed	31 (18.23)
Normal	22 (12.94)
Abnormal	9 (5.29)

The most common diagnosis during our study was febrile seizure with 92 (54.17%) patients. Neurocysticercosis was the cause of seizure in 27 (15.88%) patients, meningitis in 12 (7.05%) patients, cerebral palsy in 7 (4.11%) patients, acute encephalitis syndrome in 5 (2.94%) patients, hypoglycemia in 9 (3.4%) patients, and hyponatremia was the cause of seizure in 7 (4.11%) patients, while 2 (1.4%) patients had brain tumor. The average hospital stay was 6.28±4.03 days. The majority of patients 149 (87.65%) recovered from the illness and were discharged, 14 (8.23%) were taken against medical advice, 2 patients (1.18%) were referred to higher specialty centers, and 5 (2.94%) mortality was observed during our study. Causes of deaths are tabulated (Table 5).

**Table 5:** Final Diagnosis and Immediate Outcome (n= 170).

Variables	n (%)
**Final Diagnosis**	
Febrile seizures	92 (54.17)
Neurocysticercosis	27 (15.88)
Meningitis	12 (7.05)
Traumatic Brain Injury	9 (5.29)
Hypoglycemia	9 (5.29)
Cerebral palsy	7 (4.11)
Hyponatremia	7 (4.11)
Acute encephalitis syndrome	5 (2.94)
Brain tumor	2 (1.18)
**Immediate Outcome**	
Discharged	149 (87.65)
Leave against medical advice	14 (8.23)
Referred to higher center	2 (1.18)
Death	5 (2.94)
Acute encephalitis syndrome	2 (1.18)
Cerebral palsy	1 (0.58)
Bacterial meningitis to septic shock	1 (0.58)
Traumatic brain injury	1 (0.58)

## DISCUSSION

Seizure is one of the leading causes of hospital admission among children. In the duration of our study, 12.03% of all paediatrics admissions at our hospital presented with complaints of seizure and seizure-related conditions. This is similar to the study done by Adhikari et al, where they found that the admission rate for seizure-related illness was 12.5% which is similar to our study.^5^ Similarly, Ojha et al. in their study found the incidence to be around 10.5%, which is also similar to our study.^10^ A range of other studies have found the incidence to be as low as 3.3% to 18.3%.^6,11^

The mean age of patients in our study was 5.13±2.95 years with the majority, 104 (61.18%) of patients, being under 5 years of age. This occurrence of the first episode of seizure starting at a young age is similar to the finding of Adhikari et al. who found that 57.5% of the study population with seizure was under 5 years of age.^5^ Ojha et al. found the incidence of seizure to be higher in the under 5 years age group at 79.5%, which is higher than our study.^10^ Chen et al. in their study found a very high proportion of seizures in the under 5 years age group at 94%, which is drastically higher than ours.^7^ A higher incidence of febrile seizures must have positively impacted the outcome causing the increased prevalence of seizures in the under 5 years age group in our study. In contrast, studies done by Kumar et al. and Chaudhary et al. found the majority of cases of seizure to occur nearer to the 10-year-age group and older. The most common cause of seizures was CNS infection in Kumar's study and neurocysticercosis in Chaudhary's study.^3,6^ Both neurocysticercosis and CNS infection are more prevalent in the older age group, compared to febrile seizures which are limited to 5 years of age as prevalent in our study.^5^

The occurrence of the first episode of seizure was significantly higher in males at 72.36%. This drastic male predominance is well evidenced in the literature. Similar male predominance was seen in the studies by Chaudhary et. al at 61.9%, Adhikari et al. at 61.3%, and Ojha et al. at 65%.^5,6,10^ Chen et. al found the incidence to be greater in the male population but the proportion was almost similar between the two sexes at 54.2% for the male population and 45.8% for the female population.^7^

Generalized tonic-clonic seizure was the most common type of seizure observed during our study in 132 (77.64%) patients. A similar high incidence of generalized tonic-clonic seizure was reported by Idro et al at 78.55%, Chen et al. at 71.2%, and Adhikari et al. at 69.9%.^5,7,11^ Febrile seizures mostly present with generalized tonic-clonic seizures which could help to explain the high incidence in these studies. Idro et al. also observed that the proportion of focal seizures decreases with an increase in age, with them observing 30.2% of all seizures to be focal in the <1 month age group to only 8.9% in the 37-72 month age group.^10^ In our study, we only observed 4 cases of partial seizure. We however did not include children under 6 months of age like Idro et al. did in their study.

Fever was the most commonly associated symptom along with seizure in our study. Fever was present in 150 (88.23%) patients during our study, followed by vomiting in 68 (40%). In the study done by Adhikari et al, they also found fever to be the most common symptom associated with seizures. However, in their study, they found the occurrence to be lower at 53.53%. This must have been due to the higher prevalence of epilepsy in their study findings, which presents as unprovoked seizures without fever.^5^ In the study by Idro et al., they found the co-occurrence of fever and seizures to be as high as 92.1%, which is similar to the finding of our study.^11^ Chaudhary et al. in their study found fever to be present in only 39.9% of cases, with fever also not being the most common associated symptom. They found unconsciousness to be the most common symptom.^6^ The most common cause of the seizures in the study by Chaudhary et al. was neurocysticercosis, in which the occurrence of neurological symptoms is much more common than fever.^12^ Hence, the occurrence of fever during the seizures favors more towards the diagnosis of febrile seizures.

Chen et al. found 10.5% of children presenting with seizures to have a family history of seizures and Chung et al. found the association to be higher at 17.5%.^7,13^ Wolf et al. in their study found that 25-40% of seizures in children are associated with a family history of seizures.^14^ In our study, we found the association to be lower at 7.64%, but in our part of the world underdiagnosed and missed diagnosed cases are very common. This might have led to a smaller rate of association in our study.

We performed laboratory examinations in all the cases. Raised levels of CRP were seen in 121 (71.18%) patients. The raised CRP levels are similar to the metanalysis done by Zhong et al, where they found a positive correlation between raised CRP levels and the occurrence of seizures.^15^ In our study, only 46 (27.05%) patients had abnormal Cerebro-spinal fluid (CSF) findings. This is very similar to the findings of Adhikari et al. where they found 29.21% of the children with seizures to have abnormal CSF findings.^5^ In the study by Kumar et al., they had only done CSF analysis in 45.23% of patients, whereby they found among the analyzed cases 68.4% had abnormal CSF findings.^3^ The American Academy of Paediatrics (AAP) recommends lumbar puncture for febrile seizures in children less than 12 months of age.^9^ Thus, it should be possible to do lumbar puncture by selection of the patients through clinical symptoms to increase the sensitivity of the test and reduce the economic burden on the patient.

Neuroimaging revealed 52 (30.59%) of patients to have abnormalities. The major abnormality seen was neurocysticercosis in 27 (15.88%) of cases. In the study by Adhikari et al, they found abnormality in neuroimaging in 45.9% of children which is more than our study.^5^ Chaudhary et al found an even higher rate of abnormality in their study. They found an abnormality in neuroimaging in 56.5% of children.^6^ Both studies found neurocysticercosis to be the leading abnormality found during neuroimaging that leads to seizures in the paediatrics population.^5,6^ Neuroimaging is particularly helpful in the diagnosis of relatively obscure causes of seizures such as neurocysticercosis more so in areas such as ours where neurocysticercosis is a fairly common cause of seizures in children but is frequently underdiagnosed.

Febrile seizures were the most common cause of the first episode of seizures among 92 (54.17%) patients during our study. This finding is similar to the finding by Taherian et al., where they found febrile seizures to be the leading cause of the first episode of seizures as well.^4^ Chen et al found febrile seizures to be the cause of 62.1% of the first episode of seizure in a child, which is very similar to our study.^7^ In the study by Ojha et al., they found infection to be the leading cause of seizures in a child at 86%.^10^ Similarly, Idro et al, found infection to cause seizures in 80% of cases in their study.^11^ Chaudhary et al found neurocysticercosis to be the leading cause of seizures in their study.^6^ Adhikari et al in their study concluded seizure disorders to be the leading cause of seizures at 33.6% only then followed by febrile seizures at 30.5% of cases.^5^

Following the specific course of treatment according to the etiological profile, they were discharged from the hospital. Five (3.4%) mortalities were observed during our study. The mortality rate observed in the study by Adhikari et al. was 4.4% which is comparable to ours.^5^ The study by Idro et al in Kenya observed a mortality of 3.1% which suggests that the mortality of seizure-related disorders in children is fairly uniform despite of variance of etiology.^11^

Our study being a single-center descriptive study with a limited study pool is a relatively poor predictor for the general population. To further know the etiological and clinical profile of the occurrence of the first episode of seizure in children, a multi-institutional study should be recommended.

## CONCLUSIONS

The most common cause of the first episode of seizures in children was febrile seizure followed by neurocysticercosis and CNS infection, with generalized tonic-clonic seizure being the most common type of seizure in our study. Laboratory and radiological workup of febrile seizures is most often normal. Lumbar puncture, laboratory testing, and neuroimaging are valuable aids in the diagnosis of other obscure causes of seizures not explained by simple febrile seizures. Most children have a normal recovery but undesirable outcomes are also frequent.
